# Pharmacogenetic Association of *NOS3* Variants with Cardiovascular Disease in Patients with Hypertension: The GenHAT Study

**DOI:** 10.1371/journal.pone.0034217

**Published:** 2012-03-28

**Authors:** Xue Zhang, Amy I. Lynch, Barry R. Davis, Charles E. Ford, Eric Boerwinkle, John H. Eckfeldt, Catherine Leiendecker-Foster, Donna K. Arnett

**Affiliations:** 1 Division of Biostatistics and Epidemiology, Cincinnati Children's Hospital Medical Center, Cincinnati, Ohio, United States of America; 2 Department of Laboratory Medicine and Pathology, University of Minnesota, Minneapolis, Minnesota, United States of America; 3 School of Public Health, University of Texas-Houston, Houston, Texas, United States of America; 4 Human Genetics Center Health Science Center at Houston, Houston, Texas, United States of America; 5 Department of Epidemiology, University of Alabama at Birmingham, Birmingham, Alabama, United States of America; FuWai hospital, Chinese Academy of Medical Sciences, China

## Abstract

Nitric oxide synthase 3 (NOS3) catalyzes production of NO in the endothelium and may play a role in cardiovascular disease (CVD). We assessed the pharmacogenetic associations of three *NOS3* polymorphisms and three antihypertensive drugs with CVD outcomes. Hypertensive subjects (n = 30,280) from a multi-center, double-blind clinical trial were randomized to chlorthalidone, amlodipine, or lisinopril treatment (mean follow up, 4.9 years). Outcomes included coronary heart disease (CHD: fatal CHD and nonfatal myocardial infarction); stroke; heart failure (fatal, requiring hospitalization, or outpatient treatment); all-cause mortality; and end-stage renal disease (ESRD). Main effects of *NOS3* variants on outcome and genotype-treatment interactions were tested. For *NOS3* −690 C>T (rs3918226), a higher hazard ratio (HR) was found in minor allele carriers for CHD (CC = 1.00, CT+TT = 1.12 (95% confidence interval (CI) = 1.00–1.26), P = 0.048). For *NOS3* −922 A>G (rs1800779), a higher HR was found in minor allele carriers for heart failure (AA = 1.00, AG+GG = 1.10 (CI = 1.00–1.21), P = 0.046). Significant pharmacogenetic findings were observed for stroke and all-cause mortality. For −690 C>T, a lower HR was observed for stroke in minor allele carriers when treated with amlodipine versus lisinopril (CC = 0.85 (CI = 0.73–0.99), CT+TT = 0.49 (CI = 0.31–0.80), P = 0.04). For glu298asp G>T (rs1799983), a lower HR was observed for all-cause mortality in minor allele carriers when treated with amlodipine versus lisinopril (GG = 1.01 (CI = 0.91–1.13), GT+TT = 0.85 (CI = 0.75–0.97), P = 0.04). We observed significant associations with *NOS3* variants and CHD and heart failure and significant pharmacogenetic effects for stroke and all cause mortality. This suggests that *NOS3* variants may potentially provide useful clinical information with respect to treatment decisions in the future.

## Introduction

Hypertension is a major risk factor for cardiovascular disease (CVD), a leading cause of mortality worldwide. Control of hypertension is an important priority [1]. Despite improved treatment options, one-third of treated hypertensive patients have blood pressure above target thresholds [2]. One reason for this is that individual response to pharmacologic treatments varies; genetics may be an important determinant of this variable response. Evidence increasingly suggests genetic polymorphisms interact with antihypertensive treatments leading to different blood pressure responses and cardiovascular outcomes [3].

Given nitric oxide's (NO) role in regulating vascular function, the nitric oxide synthase 3 (endothelial cell) gene (*NOS3*) may play a role in cardiovascular pathology and individual responses to antihypertensive drugs [4]. NOS3 catalyzes the production of biological NO, a critical signaling molecule in the relaxation of vascular smooth muscle and vasodilatation. Reduction in basal NO release may predispose humans to hypertension, thrombosis, vasospasm, and atherosclerosis [5]. Conversely, overproduction of NO can also damage cells and tissues. Alteration in NO level can be caused by DNA variants that impair the function of NOS3. Many reports have indicated association of *NOS3* polymorphisms with increased occurrence of cardiovascular disease, including coronary artery disease [6], [7], [8], [9], myocardial infarction [10], [11], hypertension [12], [13], and stroke [14]. Despite its pharmacogenetic potential, there are few data regarding the impact of *NOS3* variants on the drug responses in hypertension treatments.

Amlodipine is a widely prescribed antihypertensive drug. As a calcium channel blocker, amlodipine inhibits the influx of calcium into smooth muscle cells, which is thought to be the major mechanism leading to vasorelaxation. Amlodipine also causes vasodilatation through the activation of NOS3 and subsequent production of NO [15]. Moreover, one of the pathways in NOS3 activation is calcium dependent. This evidence suggests a mechanism of amlodipine action through NOS3.

In the present study, we tested whether participants in the Genetics of Hypertension Associated Treatment (GenHAT) Study with different *NOS3* genotypes randomized to amlodipine treatment had different outcomes with regard to five CVD measures than their counterparts who were randomized to lisinopril or chlorthalidone. We sought to determine whether there was a detectable pharmacogenetic association of *NOS3* variants with CVD among those randomized to one of these three antihypertensive medications.

## Methods

### Ethics Statement

Participants recruited during the parent Antihypertensive and Lipid-Lowering Treatment to Prevent Heart Attack Trial (ALLHAT) signed informed consent documents; the GenHAT study was approved by the University of Minnesota Institutional Review Board, the University of Alabama at Birmingham Institutional Review Board for Human Use, and the University of Texas Health Science Center at Houston Committee for the Protection of Human Subjects.

### Study Population

The GenHAT study is ancillary to the Antihypertensive and Lipid-Lowering Treatment to Prevent Heart Attack Trial (ALLHAT). The GenHAT population has been previously described [16]. The present study is a subgroup analysis from GenHAT in which 30,280 participants with known genotypes of *NOS3* were analyzed. Approximately half of the participants were women (47%), and 61% of the participants were white. Participants with missing genotypes were excluded from the analysis; therefore, there were 30,269, 30,239 and 30,240 participants included in the analyses for *NOS3* −690 C>T (rs3918226), *NOS3* −922 A>G (1800779) and *NOS3* glu298asp G>T (rs1799983), respectively.

### Outcome Ascertainment

Outcomes of interest in this analysis were coronary heart disease (CHD), including fatal CHD and nonfatal myocardial infarction; stroke; heart failure (fatal, requiring hospitalization, or treated in an outpatient setting); all-cause mortality; and end stage renal disease. Outcomes were reported by clinical investigators. For outcomes involving death, documentation was obtained from death certificates and national databases to identify deaths among participants lost to follow-up. A detailed outcome ascertainment for ALLHAT has been previously published [17].

### Genotyping

DNA samples were collected on FTA paper (Fitzco Inc., Maple Plain, MN) and processed as previously described [18]. A multiplex PCR and immobilized probe-based research assay (Roche Molecular Systems, Pleasanton, CA, USA) [19] was used to genotype the three single-nucleotide polymorphisms (SNPs) listed above. Although the SNPs used in these analyses were chosen because of their availability in the Roche genotyping assay, the variants chosen by Roche were selected on the basis of disease association and functional data available at the time of the assay's design; there is evidence of association with cardiovascular disease in various ethnic and other subgroups for each of the variants [20], [21], [22]. The pairwise linkage disequilibrium R^2^ values for the 3 SNPs in the 1000 Genomes CEU population are rs3918226-rs1800779: 0.132; rs3918226-rs1799983: 0.092; rs1800779- rs1799983: 0.137. R^2^s were considerably lower in other race groups.

### Statistical Analysis

Statistical Analysis Software (SAS), version 9.1 (SAS Institute Inc., Cary, NC) was used for statistical analysis. Hardy-Weinberg (HW) equilibrium tests were performed using chi-square tests. Cox regression was used to test the main effects of *NOS3* genotypes and genotype×treatment interactions on clinical outcomes, resulting in hazard ratios (HRs). In each of the *NOS3* genotypes, the outcomes from amlodipine-treated patients were compared with those from patients treated with chlorthalidone and lisinopril using Cox regression. The main effects of genotypes on outcomes were assessed without or with adjustments for treatment, age, sex, race, Hispanic status, baseline body mass index, diabetes status, baseline total cholesterol, smoking status, and baseline systolic and diastolic blood pressures. The previously published GenHAT design paper [23] outlined six primary, *a priori* hypotheses; however, these hypotheses did not include testing the pharmacogenetic effect of *NOS3* variants. Therefore, secondary investigations such as this study are considered exploratory and, as such, are not adjusted for multiple comparisons. Since we performed multiple statistical tests of the pharmacogenetic effects of *NOS3* variants, caution must be exercised in interpreting these findings.

## Results

Baseline characteristics for the 30,280 participants are shown in Table 1. No differences were found in baseline values among treatment groups.

**Table 1:** Baseline characteristics for participants (n = 30,280) by treatment group.

Characteristic	Amlodipine	Lisinopril	Chlorthalidone	P value*
Sample size, n (%) by treatment	8,178 (27.0)	8,237 (27.2)	13,865 (45.8)	
Age (y), mean (SD)	66.9 (7.7)	66.8 (7.8)	66.8 (7.7)	0.92
Race:				
White, n (%)	4,955 (60.6)	5,000 (60.7)	8,424 (60.8)	0.85
Black, n (%)	2,834 (34.7)	2,828 (34.3)	4,741 (34.2)	
American Indian/Alaskan native, n (%)	19 (0.2)	18 (0.2)	27 (0.2)	
Asian/Pacific Islander, n (%)	96 (1.2)	85 (1.0)	169 (1.2)	
Other, n (%)	274 (3.4)	306 (3.7)	504 (3.6)	
Hispanic, n (%)	1,554 (19.0)	1,631 (19.8)	2,704 (19.5)	0.75
Women, n (%)	3,891 (47.6)	3,819 (46.4)	6,518 (47.0)	0.30
On antihypertensive treatment, n (%)	7,409 (90.6)	7,418 (90.1)	12,510 (90.2)	0.49
Blood pressure at baseline:				
All participants, mm Hg: SBP, mean (SD)	146.2 (15.7)	146.6 (15.6)	146.2 (15.7)	0.25
DBP, mean (SD)	83.9 (10.2)	84.1 (10.0)	84.1 (10.1)	0.20
Treated at baseline, mm Hg: SBP, mean (SD)	145.1 (15.6)	145.5 (15.5)	145.2 (15.7)	0.74
DBP, mean (SD)	83.3 (10.1)	83.6 (9.9)	83.5 (10.0)	0.32
Untreated at baseline, mm Hg: SBP, mean (SD)	156.5 (12.1)	156.4 (12.4)	156.1 (12.0)	0.68
DBP, mean (SD)	89.7 (9.6)	89.1 (9.3)	89.5 (9.0)	0.42
Eligibility risk factors:				
Current cigarette smoker, n (%)	1,805 (22.1)	1,803 (21.9)	3,056 (22.0)	0.95
Type 2 diabetes, n (%)	2,976 (36.4)	2,886 (35.0)	4,964 (35.8)	0.19
HDL-C<35 mg/dL, n (%)	932 (11.4)	965 (11.7)	1,661 (12.0)	0.43
LVH by electrocardiogram, n (%)	1,398 (17.1)	1,333 (16.2)	2,236 (16.1)	0.14
BMI, mean (SD)	29.8 (6.3)	29.8 (6.2)	29.7 (6.1)	0.44
Fasting glucose, mean (SD), mg/dL	122.9 (57.3)	122.4 (55.8)	123.3 (58.5)	0.63
Total cholesterol, mean (SD), mg/dL	216.8 (43.9)	215.6 (42.2)	216.2 (43.5)	0.25
HDL cholesterol, mean (SD), mg/dL	47.2 (14.7)	46.6 (14.6)	46.8 (14.9)	0.05
Fasting triglycerides, mean (SD), mg/dL	176.8 (133.0)	175.6 (138.9)	177.0 (132.5)	0.74
Serum creatinine, mean (SD), mg/dL	1.01 (0.30)	1.02 (0.29)	1.02 (0.31)	0.04
*NOS3* −690 C>T, n (%)				
CC	7,244 (88.6)	7,293 (88.6)	12,306 (88.8)	0.32
CT	897 (11.0)	905 (11.0)	1,477 (10.7)	
TT	32 (0.4)	36 (0.4)	79 (0.6)	
*NOS3* −922 A>G, n (%)				
AA	4,169 (51.1)	4,239 (51.5)	6,990 (50.5)	0.40
AG	3,202 (39.3)	3,204 (38.9)	5,566 (40.2)	
GG	786 (9.6)	787 (9.6)	1,296 (9.4)	
*NOS3* glu298asp G>T, n (%)				
GG	4,791 (58.6)	4,773 (58.0)	8,042 (58.1)	0.16
GT	2,761 (33.8)	2,852 (34.7)	4,853 (35.1)	
TT	619 (7.6)	599 (7.3)	950 (6.9)	

* test of differences between treatment groups: ANOVA for continuous variables, chi-square for categorical variables. DBP, diastolic blood pressure; HDL-C, HDL cholesterol; LVH, left ventricular hypertrophy; SBP, systolic blood pressure.

Hardy-Weinberg equilibrium was tested in each race group. *NOS3* −690 C>T genotype frequencies were in HW equilibrium in all the race groups. *NOS3* −922 A>G genotype frequencies were in HW equilibrium in white, black, American Indian/Alaskan native, and Asian/Pacific islander, but not in the “other race” group (P = 0.045). *NOS3* glu298asp G>T genotype frequencies were in HW equilibrium in all the groups except whites (P = 0.0088).

### Main Effect of *NOS3* Variants on Clinical Outcomes

Hazard ratios for main effects of the *NOS3* variants on clinical outcomes are summarized in Table 2. No association of *NOS3* variants with any clinical outcome was detected when genotypes were analyzed individually (data not shown). When minor allele carriers were combined (CT and CC for *NOS3* −690 C>T, AG and GG for *NOS3* −922 A>G, GT and TT for *NOS3* glu298asp G>T), associations were found in the adjusted models between *NOS3* −690 C>T and CHD, and *NOS3* −922 A>G and heart failure (p<0.05). Minor allele carriers of *NOS3* −690 C>T have higher risk of CHD (HR, 1.12; 95% CI, 1.00–1.26) and minor allele carriers of *NOS3* −922 A>G have higher risk to heart failure (HR, 1.10; 95% CI, 1.00–1.21 compared to their respective wild-type homozygous individuals.

**Table 2:** Main effects of *NOS3* variants on outcomes, event frequencies and rates, hazard ratios.

	*NOS3* −690 C>T			*NOS3* −922 A>G			*NOS3* glu298asp G>T		
Outcome	CC (n = 26,843)	CT+TT (n = 3,426)	P value	AA (n = 15,398)	AG+GG (n = 14,841)	P value	GG (n = 17,606)	GT+TT (n = 12,634)	P value
**CHD (primary endpoint)**									
Event frequency	2325	354		1326	1350		1513	1163	
Event rate*	18.8	22.7		18.7	19.8		18.7	20.1	
Unadjusted HR (95% CI)	1.00	1.20 (1.08–1.34)	**0.001**	1.00	1.06 (0.98–1.14)	0.15	1.00	1.07 (0.99–1.16)	0.07
Adjusted HR† (95% CI)	1.00	1.12 (1.00–1.26)	**0.048**	1.00	0.98 (0.90–1.06)	0.62	1.00	1.00 (0.92–1.08)	0.94
**Stroke**									
Event frequency	1218	139		720	638		826	531	
Event rate*	9.8	8.7		10.1	9.2		10.1	9.0	
Unadjusted HR (95% CI)	1.00	0.89 (0.75–1.06)	0.20	1.00	0.92 (0.82–1.02)	0.11	1.00	0.89 (0.80–0.99)	**0.04**
Adjusted HR† (95% CI)	1.00	0.96 (0.80–1.15)	0.63	1.00	0.97 (0.86–1.09)	0.58	1.00	0.95 (0.84–1.07)	0.38
**Heart failure**									
Event frequency	1769	243		970	1041		1176	833	
Event rate*	14.3	15.4		13.7	15.3		14.5	14.3	
Unadjusted HR (95% CI)	1.00	1.08 (0.94–1.23)	0.27	1.00	1.12 (1.02–1.22)	**0.01**	1.00	0.99 (0.90–1.08)	0.75
Adjusted HR† (95% CI)	1.00	1.04 (0.90–1.19)	0.63	1.00	1.10 (1.00–1.21)	**0.046**	1.00	0.94 (0.85–1.03)	0.20
**All-cause mortality**									
Event frequency	3761	498		2183	2069		2527	1728	
Event rate*	28.6	29.7		28.9	28.5		29.3	27.9	
Unadjusted HR (95% CI)	1.00	1.04 (0.95–1.14)	0.42	1.00	0.99 (0.93–1.05)	0.64	1.00	0.96 (0.90–1.02)	0.16
Adjusted HR† (95% CI)	1.00	1.05 (0.95–1.15)	0.37	1.00	0.99 (0.93–1.05)	0.72	1.00	0.97 (0.91–1.03)	0.32
**End-stage renal disease**									
Event frequency	366	30		214	181		264	131	
Event rate*	2.9	1.9		2.9	2.6		3.2	2.2	
Unadjusted HR (95% CI)	1.00	0.64 (0.44–0.93)	**0.02**	1.00	0.88 (0.72–1.07)	0.19	1.00	0.69 (0.56–0.85)	**0.0005**
Adjusted HR† (95% CI)	1.00	0.84 (0.57–1.23)	0.36	1.00	1.09 (0.98–1.35)	0.44	1.00	0.84 (0.67–1.06)	0.14

*per 1000 person-year.

† adjusted for treatment, age, sex, race, Hispanic status, baseline BMI, diabetes status, baseline total cholesterol, smoking status, baseline systolic and diastolic blood pressures. CHD, coronary heart disease (including fatal CHD and nonfatal myocardial infarction); CI, confidence interval; HR, hazard ratio.

### Pharmacogenetic Association (Genotype×Treatment Interactions) with Clinical Outcomes

Table 3 summarizes outcome frequencies and rates by genotype group, genotype-specific treatment effects, and the results of pharmacogenetic association tests for amlodipine versus chlorthalidone and amlodipine versus lisinopril. For pharmacogenetic tests, a significant P value for the genotype×treatment interaction indicates that the treatment effects differ by genotypes.

**Table 3:** Genotype×treatment interaction results, total events and event rates by genotype and treatment group.

		Number of events, event rates per 1000 person-years			Genotype-specific treatment effect hazard ratio (95%CI)		Genotype-by-treatment interaction P values*	
Outcome - Variant	Genotype	AML	LIS	CHL	AML vs. LIS	AML vs. CHL	AML vs. LIS	AML vs. CHL
**CHD (primary outcome)**								
−690 C>T	CC	642, 19.2	618, 18.6	1065, 18.8	1.03 (0.92–1.15)	1.02 (0.92–1.12)		
	CT+TT	89, 20.8	90, 21.2	175, 24.6	0.98(0.73–1.32)	0.85(0.66–1.09)	0.77	0.19
−922 A>G	AA	377, 19.6	345, 17.8	604, 18.8	1.10 (0.95–1.27)	1.04 (0.91–1.18)		
	AG+GG	352, 19.1	363, 20.0	635, 20.1	0.95 (0.82–1.10)	0.95 (0.83–1.08)	0.17	0.34
glu298asp G>T	GG	428, 19.3	384, 17.6	701, 19.0	1.10 (0.96–1.26)	1.02 (0.90–1.15)		
	GT+TT	303, 19.4	323, 20.6	537, 20.1	0.94 (0.81–1.10)	0.97 (0.84–1.11)	0.16	0.60
**Stroke**								
−690 C>T	CC	310, 9.1	361, 10.7	547, 9.5	**0.85 (0.73–0.99)**	0.96 (0.83–1.10)		
	CT+TT	25, 5.7	50, 11.6	64, 8.7	**0.49 (0.31–0.80)**	0.66 (0.42–1.05)	**0.04**	0.13
−922 A>G	AA	172, 8.8	221, 11.3	327, 10.1	0.78 (0.64–0.95)	0.87 (0.72–1.05)		
	AG+GG	164, 8.8	190, 10.3	284, 8.8	0.85 (0.69–1.05)	0.99 (0.82–1.20)	0.56	0.33
glu298asp G>T	GG	217, 9.7	241, 11.0	368, 9.9	0.88 (0.73–1.06)	0.98 (0.83–1.16)		
	GT+TT	118, 7.4	170, 10.7	243, 8.9	0.70 (0.55–0.88)	0.83 (0.67–1.04)	0.12	0.24
**Heart failure**								
−690 C>T	CC	582, 17.4	490, 14.8	697, 12.2	1.18 (1.05–1.33)	1.43 (1.28–1.59)		
	CT+TT	73, 17.1	72, 16.9	98, 13.6	1.02 (0.74–1.42)	1.27 (0.94–1.72)	0.40	0.47
−922 A>G	AA	317, 16.5	270, 13.9	383, 11.8	1.18 (1.01–1.39)	1.39 (1.20–1.61)		
	AG+GG	337, 18.4	292, 16.2	412, 12.9	1.14 (0.97–1.33)	1.42 (1.24–1.65)	0.75	0.80
glu298asp G>T	GG	403, 18.3	325, 14.9	448, 12.0	1.22 (1.06–1.42)	1.52 (1.33–1.74)		
	GT+TT	252, 16.1	237, 15.1	344, 12.8	1.07 (0.90–1.28)	1.27 (1.08–1.49)	0.25	0.09
**All-cause mortality**								
−690 C>T	CC	977, 27.4	1027, 28.8	1757, 29.1	0.95 (0.87–1.04)	0.94 (0.87–1.01)		
	CT+TT	135, 29.7	148, 32.4	215, 28.1	0.93 (0.73–1.17)	1.07 (0.86–1.33)	0.82	0.27
−922 A>G	AA	575, 28.0	602, 28.9	1006, 29.4	0.97 (0.86–1.08)	0.95 (0.86–1.05)		
	AG+GG	534, 27.3	572, 29.4	963, 28.6	0.93 (0.82–1.04)	0.95 (0.86–1.06)	0.61	0.96
glu298asp G>T	GG	687, 29.1	670, 28.7	1170, 29.7	**1.01 (0.91–1.13)**	0.98 (0.89–1.08)		
	GT+TT	425, 25.6	505, 30.0	798, 28.1	**0.85 (0.75–0.97)**	0.91 (0.81–1.03)	**0.04**	0.36
**End-stage renal disease**								
−690 C>T	CC	105, 3.0	102, 3.0	159, 2.7	1.02 (0.78–1.34)	1.11 (0.87–1.42)		
	CT+TT	7, 1.6	10, 2.3	13, 1.8	0.70 (0.27–1.85)	0.91 (0.36–2.29)	0.46	0.68
−922 A>G	AA	63, 3.2	59, 3.0	92, 2.8	1.07 (0.75–1.53)	1.14 (0.82–1.57)		
	AG+GG	47, 2.5	54, 2.9	80, 2.5	0.85 (0.58–1.26)	1.01 (0.70–1.44)	0.40	0.63
glu298asp G>T	GG	77, 3.4	73, 3.3	114, 3.0	1.04 (0.75–1.43)	1.12 (0.84–1.50)		
	GT+TT	35, 2.2	39, 2.4	57, 2.1	0.90 (0.57–1.43)	1.05 (0.69–1.60)	0.63	0.80

*P value: minor allele carriers combined into one group due to low numbers of events in some cells, H_o_ = interaction coefficient equals zero (1-degree of freedom test). AML, amlodipine; CHD, coronary heart disease (including fatal CHD and nonfatal myocardial infarction), CHL, chlorthalidone; CI, confidence interval; LIS, lisinopril.

Our data suggested a pharmacogenetic association for the −690 C>T variant with stroke, and for the glu298asp G>T with all-cause mortality when comparing amlodipine with lisinopril. When treated with amlodipine vs. lisinopril, the minor allele carriers at the *NOS3* −690 locus showed lower risk of stroke (HR, 0.49 vs. 0.85; 95% CI, 0.31–0.80 vs. 0.73–0.99; P = 0.04), and the minor allele carriers at the glu298asp locus showed lower risk of all-cause mortality (HR, 0.85 vs. 1.01; 95% CI, 0.75–0.97 vs. 0.91–1.13, P = 0.04) compared to their respective wild type homozygous individuals.

## Discussion

NO is a critical signaling molecule in many physiological and pathological processes. Three NO synthase (NOS) enzymes have been identified, which catalyze the production of biological NO from L-arginine. Primarily expressed in endothelia, the NOS3 enzyme has been proposed to be the most relevant NOS in vasodilatation and vascular diseases. Sequence variations have been reported since the cloning of *NOS3*; variations may result in reduced or excessive production of NO and contribute to cardiovascular diseases [24]. In the present study, we examined the association of the *NOS3* variants −690 C>T, −922 A>G, and glu298asp G>T with five CVD outcomes. Association was detected between −690 C>T and CHD, −922 A>G and heart failure. We also compared the pharmacogenetic effects in amlodipine group versus lisinopril and chlorthalidone groups. Our data suggest that minor allele carriers for −690 C>T and glu298asp G>T may have lower risk of stroke and all-cause mortality when using amlodipine as compared to lisinopril.

As one of the polymorphisms in the coding region of *NOS3*, the glu298asp G>T variant has been studied extensively. This G-to-T transversion at nucleotide position 894 within exon 7 results in a change of glutamate to aspartate at position 298 in the oxygenase domain of the NOS3 protein. Associations between the glu298asp G>T polymorphism and NO synthesis [25], [26] and endothelial function [27], [28] have been previously described. The change of glutamate to aspartate may affect the interaction of NOS3 with caveolin-1, thereby affecting the localization of NOS3 and, eventually, diminishing the activation of NOS3 [29]. Molecular studies suggested that, even though intact NOS3 Asp298 has equivalent enzymatic activity to NOS3 Glu298, carriers of NOS3 Asp298 may be at different disease risk if exposed to adverse environmental influence on endothelial function [30]. Epidemiologic studies have associated this polymorphism with the development of hypertension [31], CHD, and endothelial dysfunction [28]. Glu298asp G>T may also play a role in renal function. GG carriers were reported to have lower mean arterial pressure and an augmented glomerular filtration rate than the homozygotes for the wild type allele [32]. In response to a graded L-arginine infusion, the GG carriers had significant changes in effective renal plasma flow, glomerular filtration rate, filtration fraction, renal vascular resistance, and renal blood flow. In contrast, the renal response to L-arginine in GT/TT carriers was blunted. Epidemiologic studies, however, reported inconsistent results on the association between glu298asp and end-stage renal disease. This may be partly due to different cohorts chosen in different studies. Although several studies suggested the T allele is the risk allele for end-stage renal disease [33], [34], [35], [36], others reported that homozygosity for the G allele was associated with increased risk with diabetic nephropathy [37], or showed no association with ESRD [38]. Our study did not find statistical evidence that the T allele was associated with a lower risk of end-stage renal disease in hypertensive patients.

Variations in the promoter regions of *NOS3* have also been identified, among which are −922A/G and −690C/T. Although these variations are not located in the catalytic site or consensus sequences for transcription factor binding [24], [39], they may affect the protein level and enzyme activity through regulation of gene expression, thereby affecting the plasma NO metabolite levels [40], blood pressure, and risk of cardiovascular diseases. A recent genome-wide association study of essential hypertension singled out −690 C>T as potentially playing a role in hypertension. Not only were hypertension associations with the polymorphism genome-wide significant in a 2-stage case-control study, the finding was confirmed in a 21,714-subject meta-analysis. Using PATCH, the authors identified a putative binding site for ETS family transcription factors directly next to the −690 C>T locus. ETS-1 and ELF-1 are essential factors for activation of the *NOS3* promoter, suggesting that this variant might modulate the transcription of *NOS3* [41]. Our study showed that minor allele carriers for −690 C>T have higher risk in CHD, and minor allele carriers for −922 A>G have higher risk in heart failure (Table 2). The −786 T>C (rs2070744) polymorphism is the most studied variant in *NOS3* promoter. The C allele has been associated with reduced promoter activity and gene transcription [42] and may influence the risk of cardiovascular events [6], [43], cardiovascular mortality [44], and hypertension and CVD in renal allograft recipients [45]. This SNP is in strong linkage disequilibrium with −922A>G (In 1000 Genomes sample, R^2^ = 0.967 in CEU, R^2^ = 1.00 in YRI.) It is not clear if these two variants affect the risk of CVD through altering the *NOS3* transcription and NO level. More functional studies are needed to elucidate the mechanisms of −690 C>T and −922 A>G in the development of heart disease outcomes.

In the present study, we also tested whether subjects with different *NOS3* genotypes randomized to the amlodipine had different outcomes than their counterparts who were randomized to lisinopril or chlorthalidone. Significant genotype×treatment interactions were observed for stroke and all-cause mortality when comparing amlodipine to lisinopril. CC and TT genotypes for −690 C>T have a lower risk of stroke, while GT and TT genotypes for glu298asp have a lower risk of all-cause mortality. Because *NOS3* glu298asp G>T genotype frequencies were not in HW equilibrium in whites (P = 0.0088), we further stratified the white group into Hispanic whites and non-Hispanic whites and found HW equilibrium P values were 0.49 and 0.004, respectively. The all-cause mortality interaction ratio of hazard ratios (and P values) for the amlodipine versus lisinopril comparison for this variant for the combined race analysis (as reported in Table 3), the non-Hispanic whites, and the Hispanic whites were 0.84 (P = 0.04), 0.79 (P = 0.04), and 0.88 (P = 0.35), respectively. This suggests the lack of HW equilibrium in the non-Hispanic white group is not responsible for the overall pharmacogenetic association. The randomized design of the trial minimizes admixture as a possible confounder (i.e., degree of admixture would be the same in treatment groups).

It has been reported that amlodipine may cause vasodilatation through the activation of NOS3 [15]. It is known that one of the pathways in NOS3 activation is calcium dependent. Since amlodipine blocks the efflux of Ca, it may also deactivate NOS3 through Ca regulation. How these two actions of amlodipine on NOS3 interact is unknown. In our analyses of CVD outcomes, minor allele carriers tend to have more favorable outcomes when randomized to amlodipine versus lisinopril, but not amlodipine versus chlorthalidone, which suggests *NOS3* variants may be useful in some, but not all, antihypertensive treatment decisions.

For our suggestive pharmacogenetic findings, we used linear regression to determine if there was an equivalent pharmacogenetic association with change in systolic blood pressure and change in diastolic blood pressure (ΔSBP, ΔDBP respectively; that is, blood pressure at randomization minus blood pressure 6 months after randomization) for the stroke/*NOS3* −690 C>T and all-cause mortality/*NOS3* glu298asp G>T findings. This could suggest a mechanistic pathway with which to explain the interaction for outcome events. There was, however, no equivalent association (P = 0.77 and P = 0.78 for ΔSBP and ΔDBP, respectively, for the stroke findings; P = 0.07 and P = 0.59 for ΔSBP and ΔDBP, respectively, for the all-cause mortality findings).

By using a large cohort of hypertensive patients, our study suggested pharmocogenetic associations of *NOS3* variants with CVD outcomes, and demonstrated the importance of genetic information in individualized therapy.

Because GenHAT's parent ALLHAT population included only older, hypertensive participants with other risk factors for CVD, caution must be used in generalizing these findings to younger, healthier populations. In addition, because we interrogated the 23,529-base pair *NOS3* at only 3 SNP loci, this study is not a complete evaluation of the pharmacogenetic effects of *NOS3*. Although it is plausible that the polymorphisms investigated here directly influence vascular function, variants with main or pharmacogenetic effects cannot be assumed causal for CVD outcomes. Because we performed multiple tests of pharmacogenetic effects, these findings would not meet the strictest threshold of statistical significance if corrected for multiple testing (e.g., Bonferroni correction: 0.05/30 tests would equate to a P value of 0.0017).

### Conclusions

Findings from this randomized, double-blind clinical trial suggest that there are associations between *NOS3* variants and CHD and heart failure, as well as pharmacogenetic associations for the −690 C>T variant of *NOS3* with stroke, and for the glu298asp G>T variant with all-cause mortality when comparing amlodipine with lisinopril. The pharmacogenetic comparison of amlodipine versus chlorthalidone reported here suggests that consideration of these *NOS3* variants does not impinge upon ALLHAT's general recommendation of chlorthalidone as a first-step therapy. However, our suggestive pharmacogenetic associations argue for future genetic and functional studies to confirm our findings and explore the implications of *NOS3* genetic variation in hypertension treatment with respect to cardiovascular outcomes.
